# Automated enumeration and phenotypic characterization of CTCs and tdEVs in patients with metastatic castration resistant prostate cancer

**DOI:** 10.1038/s41391-020-00304-1

**Published:** 2020-11-23

**Authors:** Steffi Oeyen, Vincent Liégeois, Bram De Laere, Andy Buys, Michiel Strijbos, Piet Dirix, Paul Meijnders, Peter Vermeulen, Steven Van Laere, Luc Dirix

**Affiliations:** 1 Center for Oncological Research (CORE), Faculty of Medicine and Health Sciences, University of Antwerp, Antwerp, Belgium; 2 Translational Cancer Research Unit (TCRU), GZA Hospitals Sint-Augustinus, Antwerp, Belgium; 3 Faculty of Medicine and Health Sciences, University of Antwerp, Antwerp, Belgium; 4 Department of Medical Epidemiology and Biostatistics, Karolinska Institute, Stockholm, Sweden; 5 Department of Human Structure and Repair, Ghent University, Ghent, Belgium; 6 Department of Medical Oncology, GZA Hospitals Sint-Augustinus, Antwerp, Belgium; 7 Iridium vzw, Antwerp, Belgium

**Keywords:** Prognostic markers, Outcomes research, Prostate cancer, Translational research, Prognostic markers

## Abstract

**Background:**

Although most patients with metastatic castration-resistant prostate cancer (mCRPC) initially benefit from treatment with androgen receptor signaling inhibitors (ARSi), resistance inevitably occurs. Hence, we investigated the prognostic value of automated circulating tumor cell (CTC) and tumor-derived extracellular vesicle (tdEV) enumeration and their dynamics, in patients with mCRPC in the context of the initiation of treatment with ARSi. Furthermore, we hypothesize that CTC phenotypic heterogeneity might serve as a measurable biomarker under these circumstances.

**Methods:**

Using an image analysis tool, we reanalyzed all CellSearch images previously acquired in the context of a prospective, multicenter clinical study for patients with mCRPC (*n* = 170) starting a new line of ARSi, for CTC and tdEV detection and enumeration. CTC (*n* = 19 129) phenotypic diversity was quantified by the Shannon index (SI). Progression-free survival (PFS) and overall survival (OS) were compared between groups of patients stratified according to CTC, tdEV, and SI levels.

**Results:**

Automated CTC enumeration provided similar clinical prognostication compared with operator-based counts. Patients demonstrating high CTC phenotypic heterogeneity before therapy had a shorter median PFS (4.82 vs. 8.49 months, HR 1.79; *P* = 0.03) and OS (12.6 months vs. not reached, HR 2.32; *P* = 0.03), compared to patients with low diversity, irrespective of CTC level. Multivariable analysis showed how the prognostic value of the baseline SI was lost by pretreatment chemotherapy status, CTC counts, and PSA levels.

**Conclusions:**

Automated CTC counts are a reliable substitute for reviewer-based enumeration, as they are equally informative for prognosis assessment in patients with mCRPC. Beyond enumeration, we demonstrated the added value of studying CTC phenotypic diversity for patient prognostication, warranting future investigation.

## Introduction

One of the major hurdles in the treatment of patients with advanced prostate cancer (PCa) is spatial and temporal tumor heterogeneity, leading to fluctuating responses and acquired therapy resistance. Circulating tumor cells (CTCs), as a real-time snapshot of the prevailing disease, can provide vital information for evaluating (sub)clonal mutational patterns and monitoring the evolution thereof [1].

CTC detection and enumeration by means of the FDA-cleared CellSearch platform (Menarini, Silicon Biosystems, Italy) can be used as a prognostic biomarker before and during treatment [2–4]. Although CTC counts can be used to evaluate therapy response [4, 5], management based on CTC changes has not been demonstrated. Interestingly, apart from CTCs, other EpCAM+/Cytokeratin (CK)+ objects, potentially carrying prognostic information, exist [6]. In addition, enumerating fusion hybrids of neoplastic cells and leukocytes demonstrated a correlation with disease stage and OS [7]. Furthermore, EpCAM+/CK+/CD45− small and large micro-sized tumor fragments, both positive and negative for nuclear content, are related with poor outcome in metastatic castration-resistant prostate cancer (mCRPC) [6]. Enumeration of such particles without a nucleus, hereinafter referred to as tumor-derived extracellular vesicles (tdEVs), provided similar prognostic relevance to CTC counts [8].

During a prospective, multicenter clinical study we previously detected traditional CTCs in up to 70% of our ‘all-comer’ mCRPC cohort prior to androgen receptor signaling inhibitors (ARSi) [9]. Here, we reanalyzed all acquired CellSearch images, using the open source automated CTC Classification Enumeration and PhenoTyping (ACCEPT) tool. We reassessed the prognostic value of automated CTC and tdEV enumeration in mCRPC patients prior to and during treatment, and upon disease progression. In addition to enumeration, characterization of phenotypic heterogeneity on a single CTC level would allow for a better understanding of malignant progression and the possible identification of phenotypic resistance biomarkers. Along these lines, it was recently shown that low phenotypic heterogeneity in the CTC compartment of mCRPC patients was associated with improved overall survival (OS) in the context of treatment with pathway-specific hormonal agents, whereas patients with a high CTC phenotypic heterogeneity demonstrated more therapeutic benefit from treatment with taxane-based chemotherapy [10, 11]. Therefore, we retrospectively studied the phenotypic diversity in the CTC population prior to treatment, and its association with outcome, in patients with mCRPC in the context of ARSi. This heterogeneity was quantified by the Shannon’s diversity index (SI), a popular diversity index in the field of ecology.

## Materials and methods

A detailed description of materials and methods is provided in Supplementary Materials and Methods. In brief, peripheral blood sample collection from patients with mCRPC, and CTC detection and enumeration were performed as described previously [9]. All archived CellSearch images were reanalyzed using the image analysis tool ACCEPT. CTC and tdEV counts and phenotypic properties were extracted from the software and used to perform extensive tdEV and phenotypic CTC analysis.

## Results

### Patient and sample characteristics

In total, 331 CellSearch image libraries from 170 unique mCRPC patients were available for analyses, of which 143 at baseline (43%), 114 at 10–12 weeks follow-up (34%), and 74 at disease progression (22%). Their average age at registration was 75 years. Patient characteristics and baseline blood chemistry measures are listed in Supplementary Tables S2 and S3, respectively. The majority of patients did not receive any ARSi or chemotherapy for mCRPC prior to inclusion (86% and 62%, respectively). While 71% (120/170) of patients initiated abiraterone acetate, only 29% (50/170) started treatment with enzalutamide at baseline for mCRPC.

### CTC and tdEV counts

**Fig. 1:**
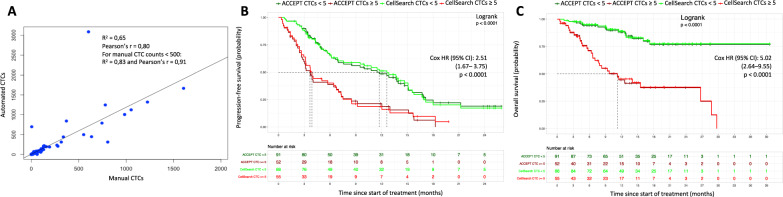
Relation between manual and automated CTC counts. **A** Depicts a scatterplot showing a linear relationship between operator-based and software-based CTC counts. Kaplan–Meier estimates of progression-free (**B**) and overall survival (**C**) for baseline CTC counts of our studied patient cohort. Patients were stratified according to their baseline CTC levels <5 or ≥5 CTC/7.5 mL into favorable (green curves) and unfavorable (red curves) groups of patients, respectively. The automated CTC counts are represented by dark colors, the reviewer-based CTC counts by bright colors. CTCs circulating tumor cells, HR hazard ratio, CI confidence interval.

At study entry 98/143 patients (69%) were CTC positive (≥1 CTC). Results reveal a linear relationship between manual (reviewer-based using CellSearch criteria) and automated (ACCEPT-based) CTC counts, particularly in the lower range of counts, with augmenting variability as CTC levels increase (Fig. 1A). The number of automated CTCs was significantly correlated with the number of tdEVs (Supplementary Fig. S1A). tdEV counts (Median, range: 16, 0–10951), however, are generally 16-fold higher as compared to both automated (Median, range: 1, 0–3088) and manual (Median, range: 1, 0–1993) CTC counts.

Both automated CTC and tdEV counts decreased significantly post treatment, after 10–12 weeks follow-up, and again increased in patients demonstrating progressive disease (Supplementary Fig. S2). In addition, whereas patients that were chemotherapy pretreated showed significantly higher automated CTC and tdEV levels at baseline compared to patients that were chemotherapy naïve, patients previously exposed to ARSi, as compared to patients that were not treated with ARSi prior to study entry, did not (Supplementary Fig. S3).

### CTCs and tdEVs vs. survival

Next, patients were dichotomized according to baseline CTC (<5 or ≥5 CTCs/7.5 mL) and tdEV (<105 or ≥105 tdEVs/7.5 mL) levels into favorable and unfavorable groups, respectively, as described and validated elsewhere [2, 8]. Patients with baseline automated CTC measures ≥5 CTCs/7.5 mL (*n* = 52), as compared to patients with <5 CTCs/7.5 mL (*n* = 91), had a shorter median PFS (3.7 vs. 11.7 months, hazard ratio (HR) 2.51, 95% confidence interval (CI) 1.67–3.75; *P* < 0.0001) and OS (11.2 months vs. not reached, HR 5.02, CI 2.64–9.55; *P* < 0.0001), recapitulating patient prognostication with striking similarity when compared to manual CTC enumeration (Fig. 1B, C).

Besides CTCs, the presence of high baseline tdEV levels (≥105 tdEVs/7.5 mL) was strongly correlated with poor clinical outcome. Patients with unfavorable baseline tdEV measures (*n* = 37), as compared to patients with favorable levels (*n* = 106), had a shorter median PFS (3.21 months vs. 10 months, HR 2.42, 95% CI 1.56–3.73; *P* < 0.0001) and OS (7.74 months vs. 29.74, HR 4.52, CI 2.45–8.34; *P* < 0.0001) (Supplementary Fig. S1B, C, respectively).

In our previous study, we provided evidence that not only high CTC levels at baseline, but also CTC dynamics during therapy can provide independent clinical prognostication over PSA declines [9]. Therefore, patients were classified based on automated CTC dynamics, as having either equal (stable), increasing or decreasing CTC counts in their follow-up as compared to baseline samples. Patients demonstrating increasing automated CTC counts (*n* = 17), as compared to patients with decreasing (*n* = 42) or stable (*n* = 35) CTC counts during therapy, had a shorter median PFS (4.59 vs. 12.85 vs. 15.08 months, HR 2.96, CI 1.48–5.89; *P* = 0.002), and OS (12.8 vs. 26.7 months vs. not reached, HR 4.01, CI 1.50–10.73; *P* = 0.006) (Supplementary Fig. S4).

### Phenotypic diversity in the CTC compartment

**Fig. 2:**
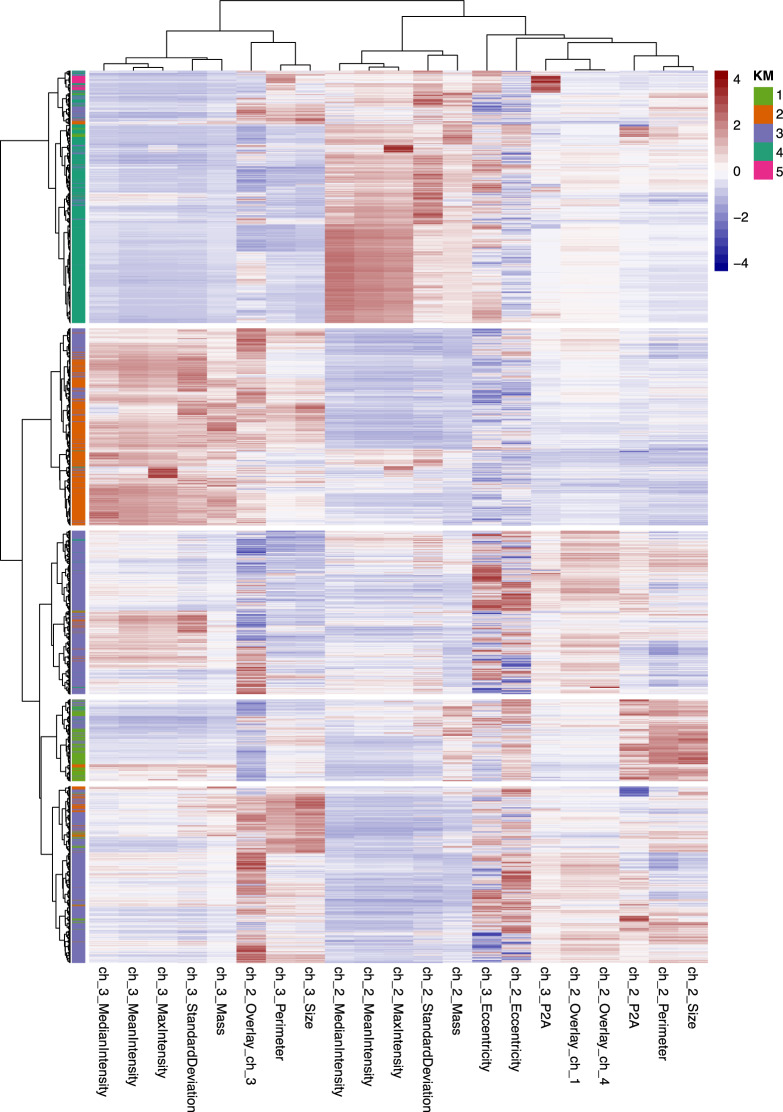
Heatmap depicting the results from an unsupervised hierarchical clustering analysis of the phenotypic properties of 19.129 CTCs. Each column shows a phenotypic parameter (ch_2 and ch_3 represent the measures acquired for the nuclear channel (DNA) and cytokeratin channel, respectively), each row a CTC. KM k-means cluster.

Applying k-means clustering, based on their phenotypic properties provided by the ACCEPT algorithm, the CTCs were partitioned into five clusters. Parameters driving the clustering pattern are shown in the heatmap depicted in Fig. 2 and Supplementary Fig. S5. To provide evidence that these clusters reflect genuine CTC phenotypes and are not just driven by those samples with exceedingly high CTC counts, Euclidean distances were calculated between each CTC and the centroids of their assigned cluster. In case the clustering pattern is directed by samples with high CTC counts, it stands to reason that these CTCs are closer to the cluster centroids resulting in smaller Euclidean distance values. However, CTC-to-centroid distances observed in samples with high and low CTC counts were not different (Supplementary Fig. S6), indicating that CTCs from both sample groups are equally well represented by the clusters.

**Fig. 3:**
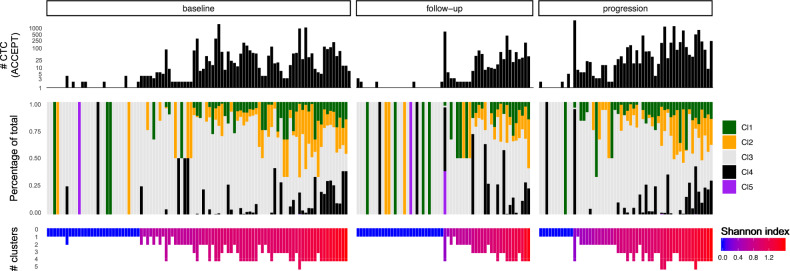
Phenotypic CTC cluster distributions per patient sample ranked according to SI level partitioned per sample type (baseline, follow-up and progression) in CTC-positive patients (*n* = 211, 64%). The percentage of a certain cluster is illustrated by the colored bars in the middle panel. CTCs circulating tumor cells, SI Shannon index, Cl cluster.

Whereas CTCs from cluster 3 are present in almost all patient samples, the presence of CTCs from cluster 5 is a rather rare event (Fig. 3 and Supplementary Fig. S7). CTCs residing in cluster 1 are characterized by elevated values for parameters concerning the size and shape of the DAPI signal, indicating that this cluster holds cells with large, rather oval nuclei. CTCs in cluster 2 are larger CTCs and feature high CK signal intensities. The fourth cluster contains smaller CTCs with an intense, relatively small DAPI signal and low CK signal. During treatment, the general number of different CTC phenotypic clusters increased.

In patients that were CTC positive at baseline, we revealed that the presence of CTCs from clusters 1, 2, and 4 was associated with worse PFS (Logrank test, all *P* < 0.05). In contrast, the presence of CTCs belonging to cluster 3 or 5 was not indicative for prognosis (Supplementary Fig. S8). Patients with cluster 1 (*n* = 52), cluster 2 (*n* = 50), or cluster 4 (*n* = 40) CTCs, had a significantly shorter median PFS compared to patients that did not have any CTCs in cluster 1 (*n* = 46, 3.9 vs. 7.31 months, HR 1.77, CI 1.18–2.66; *P* = 0.006), 2 (*n* = 48, 3.7 vs. 6.98 months, HR 1.81, CI 1.2–2.74; *P* = 0.005), or 4 (*n* = 58, 4.59 vs. 6.66 months, HR 1.85, CI 1.21–2.83; *P* = 0.004). Although according to the univariable (UV) cox proportional hazards analysis the presence of CTCs from cluster 3 was significantly associated with PFS (*P* = 0.041), most patients had CTCs belonging to this category causing insufficient numbers of patients in the negative category.

Based on the division of CTCs into various phenotypic categories the SI was calculated. Results revealed a weak, insignificant, and positive correlation between the SI and CTC counts (*r* = 0.19, *P* = 0.004). The diversity index decreased significantly after 10–12 weeks of endocrine therapy (*P* = 0.004), and again increased in patients that developed therapy resistance (*P* = 0.0001). Furthermore, chemotherapy pretreated patients demonstrated a higher SI at baseline as compared to chemotherapy-naïve patients (*P* = 0.029). In contrast, patients that were already exposed to ARSi before the start of the study showed no difference in their SI compared to patients that were not previously exposed to ARSi (*P* = 0.51).

**Fig. 4:**
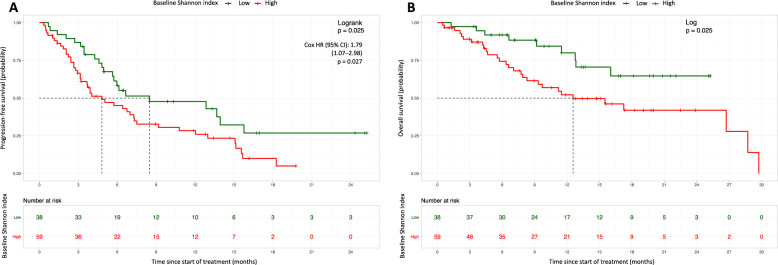
Shannon index vs. survival. Kaplan–Meier estimates (probabilities) of progression-free (**A**) and overall survival (**B**) in relation to the baseline Shannon index. Patients were stratified according to their baseline Shannon index values (<0.68 or ≥0.68), into favorable (green curves) and unfavorable (red curves) groups of patients, respectively. HR hazard ratio, CI confidence interval.

Figure 4 demonstrates that a high SI correlates with poor clinical outcome, as patients with a high index showed a shorter median PFS (4.82 vs. 8.49 months, HR 1.79, CI 1.07–2.98; *P* = 0.027) and OS interval (12.6 months vs. not reached, HR 2.32, CI 1.09–4.96; *P* = 0.029), compared to patients with a low value. Multivariable (MV) cox proportional hazards regression analysis based on CTC counts, previously and newly defined baseline variables, significantly associated with PFS in UV analysis, revealed that chemotherapy pretreatment (HR 2.832, CI 1.467–5.467; *P* = 0.002), baseline PSA values (HR 1.001, CI 1.000–1.002; *P* = 0.018), and baseline CTC counts (HR 1.003, CI 1.002–1.004; *P* < 0.0001) were independently associated with a shorter PFS (72 patients were excluded due to missing values, Table 1). Prior chemotherapy exposure (HR 3.372, CI 1.532–7.423; *P* = 0.003), baseline PSA values (HR 1.002, CI 1.000–1.003; *P* = 0.009) and CTC counts at baseline (HR 1.006, CI 1.002–1.010; *P* = 0.003) were independently predictive for OS (Table 1). Building a separate model, MV analysis based on tdEV counts, previously and newly defined baseline variables, revealed that the prognostic value of baseline PSA measures was outperformed by baseline chemotherapy status and tdEV counts (Supplementary Table S4). Concordance indices (C-indices) between both models (for CTC and tdEV counts) were compared and showed slightly higher, but substantially similar, C-indices for the tdEV model (PFS 0.734 vs. 0.723 and OS 0.768 vs. 0.763 for models based on tdEV and CTC counts, respectively). In MV analysis the prognostic effect of the SI was outdone by CTC counts and other factors for prognosis assessment. In addition, a sensitivity analysis on baseline samples from chemotherapy- and ARSi-naïve patients (*n* = 87) was performed. A model for both the CTC (Supplementary Table S5) and tdEV (Supplementary Table S6) variables separately was developed. In MV analysis, both baseline CTC as tdEV values remain predictive for PFS, however, their predictive value is insignificant for OS.

**Table 1:** Uni- and multivariable Cox proportional hazards regression analysis of progression-free (A) and overall (B) survival based on CTC counts, previous and newly defined baseline characteristics.

		Univariable		Multivariable	
		Cox proportional hazard		Cox proportional hazard	
Variable	Categories	HR (95% CI)	*P*^a^	HR (95% CI)	*P*^a^
(A) PFS					
Age	Continuous	1.018 (0.993–1.043)	0.159		
Chemotherapy status	Naïve vs. pretreated	2.601 (1.729–3.912)	**<0.0001**	2.832 (1.467–5.467)	**0.002**
Prior ARSi exposure	No vs. yes	1.715 (1.011–2.909)	**0.045**	1.030 (0.444–2.388)	0.945
Metastases at start of study	Non-visceral/nodal vs. visceral	2.402 (1.459–3.953)	**0.001**	0.938 (0.478–1.842)	0.852
Baseline PSA	Continuous	1.002 (1.001–1.002)	**<0.0001**	1.001 (1.000–1.002)	**0.018**
Baseline LDH	Continuous	1.001 (1.001–1.002)	**<0.0001**	1.000 (1.000–1.001)	0.528
Baseline CTC (/7.5 mL)	Continuous	1.003 (1.002–1.005)	**<0.0001**	1.003 (1.002–1.004)	**<0.0001**
Baseline SI	Continuous	1.918 (1.196–3.075)	**0.007**	1.701 (0.812–3.563)	0.159
(B) OS					
Age	Continuous	1.008 (0.971–1.048)	0.671		
Chemotherapy status	Naïve vs. pretreated	3.567 (1.875–6.786)	**0.0001**	3.372 (1.532–7.423)	**0.003**
Prior ARSi exposure	No vs. yes	1.853 (0.884–3.884)	0.102		
Metastases at start of study	Non-visceral/nodal vs. visceral	2.879 (1.509–5.493)	**0.001**	1.657 (0.741–3.704)	0.219
Baseline PSA	Continuous	1.002 (1.001–1.003)	**<0.0001**	1.002 (1.000–1.003)	**0.009**
Baseline LDH	Continuous	1.001 (1.0004–1.002)	**0.001**	0.999 (0.997–1.000)	0.053
Baseline CTC (/7.5 mL)	Continuous	1.003 (1.002–1.004)	**<0.0001**	1.006 (1.002–1.010)	**0.003**
Baseline SI	Continuous	3.034 (1.479–6.228)	**0.002**	1.901 (0.748–4.830)	0.177

*PFS* progression-free survival, *OS* overall survival, *HR* hazard ratio, *CI* confidence interval, *CTC* circulating tumor cells, *PSA* prostate-specific antigen, *LDH* lactate dehydrogenase, *SI* Shannon index.

Bold values indicate statistical significance *p *< 0.05.

^a^*P* values from Wald test of Z statistic.

## Discussion

Historically, the main focus was directed towards the CTC enumeration and prognostication [2, 9]. However, as treatment options for patients with mCRPC are expanding expeditiously, the need for sensitive predictive biomarkers, imperative in precision medicine, rises along. Therefore, the past few years, the potential of CTCs for biomarker assessment has been explored in greater detail [12]. We previously reported a prospective clinical cohort study in mCRPC which demonstrated that transcriptional profiling of CTCs for AR splice variants is feasible, identifying a subset of patients with poor outcome on ARSi [9, 13, 14]. Here, we performed a post hoc analysis on acquired CTC image data and demonstrate that phenotypic properties of CTCs, and therapy dependent changes thereof, can also be exploited for clinical decision-making.

Using previously established gating settings for CTC and tdEV detection by Nanou et al. [8], we subjected 331 image libraries from 170 patients to analysis. A strong correlation between automated ACCEPT-based and manual operator-based CTC counts was observed, resulting in identical PFS and OS estimates. This warrants CTC enumeration amenable to standardization, eliminating both inter- and intra-reviewer variation and allowing for more reliable between-study comparisons. Besides CTCs, ACCEPT-based enumeration of tdEVs revealed a strong correlation with CTC numbers, as reported [8]. Enumeration of tdEVs, that are up to 16-fold more prevalent, provided similar prognostic information as compared to CTCs, making tdEVs a promising and possibly more sensitive treatment monitoring biomarker. Moreover, in MV analyses, we again observed how tdEV counts provided similar prognostic value compared to CTC counts. In comparison to the entire cohort, the prognostic value of CTCs and tdEVs was again recapitulated in chemotherapy- and ARSi-naïve patients for PFS, but not OS, which might be in part explained given the low number of OS events at the time of database lock. However, since the CellSearch system is designed to analyze the tumor cell fraction, and plasma is aspirated during preprocessing, it should be emphasized that the CellSearch-derived tdEV counts are an underestimation of the true vesicle count. As tdEVs are considered to be a mechanism for intercellular communication supporting tumor progression, future research should be aimed at unraveling the composition and exact origin of these abundant subcellular objects [15–18].

In addition to enumeration, ACCEPT permits a more detailed analysis of the image libraries by extracting various features from the fluorescent images. We observed that CTCs in our mCRPC cohort were phenotypically heterogeneous both within and between patients, which was mainly attributable to differences in nuclear size, nuclear content, and CK intensities.

Although we solely explored strictly defined CTCs, and thereby only CK+ cells, our data revealed the existence of cells with attenuated values for features related to CK expression (i.e., cluster 4), which has clinical relevance as patients carrying this type of cells demonstrated significantly shorter PFS compared to noncarriers. Varying CK expression in CTCs has been reported previously, with cells having decreased or no CK expression being associated with worse OS [19, 20]. These cells might have undergone epithelial–mesenchymal transition, which can contribute to the acquisition of stem cell features leading to an acquired therapy resistance phenotype and possibly facilitating metastasis formation [21, 22]. Interestingly, CTCs from patients with neuroendocrine differentiation in PCa, a treatment-emergent subtype associated with androgen deprivation therapy resistance, exhibited low CK expression and smaller cell morphology, as we observed as well in cluster 4-positive CTCs [23].

In order to quantify the extent of phenotypic heterogeneity per sample, the SI was calculated based on the number of CTCs per CTC cluster per sample. SI dichotomization revealed that an elevated SI was associated with adverse prognosis, which was maintained when the SI was used as a continuous variable in time-to-event analysis. Importantly, no correlation between CTC count per sample and SI was observed. Thereby providing evidence of the added value of studying CTC phenotypic heterogeneity for patient prognostication. Similar findings were reported by Scher et al., which supports the hypothesis that baseline CTC phenotypic diversity, may predict ARSi outcome, and again by Armstrong et al., who prospectively evaluated the SI and additionally saw that high CTC heterogeneity, was more common in AR splice variant 7 (AR-V7) positive patients [10, 11]. However, MV analysis demonstrated how the prognostic value of the SI was outperformed by CTC counts and clinical features (i.e., PSA and prior chemotherapy exposure), warranting future optimization of the phenotypic diversity measurement (i.e., amongst others; targeting more tumor cell proteins in order to increase the number of parameters available for analysis).

Interestingly, we observed heterogeneous dynamics in CTC phenotypic diversity during the course of therapy, typically (but not always) characterized by a decrease in SI at follow-up, which eventually increased at progressive disease (Supplementary Fig. S7). A potential hypothesis for this decline in diversity might be that ARSi potentially targets a specific subpopulation of CTCs, thereby reducing phenotypic variation in the CTC compartment. Towards progression the diminished subpopulation might have become resistant to the therapy, resulting in CTC expansion, accompanied with increased diversity. The number of different CTC clusters increased, often even in patients that were clinically responsive at follow-up (Supplementary Fig. S7). Perhaps, these patients were deemed clinically and radiologically not progressive at the moment of second-time sampling, whereas evidence of treatment resistance was already unfolding in the bloodstream, as all these patients eventually acquired progressive disease. These data indicate that monitoring phenotypic CTC diversity during patient follow-up may provide early indications of disease progression.

In conclusion, automated analysis of CTCs using the ACCEPT software is a promising step towards standardized CTC scoring and phenotypic characterization, however, its application possibilities are copious. When quantified by e.g., SI, CTC phenotypic heterogeneity harbors prognostic value, irrespective of the CTC level, in ARSi-treated patients with mCRPC. During therapy, CTC phenotypic heterogeneity is subjective to change and could serve as pharmacodynamic biomarker, warranting future clinical investigation.

## Supplementary information

Supplementary Material and Methods

Supplementary Figures

Supplementary Tables
