# Supplementary material for: Automated enumeration and phenotypic characterization of CTCs and tdEVs in patients with metastatic castration resistant prostate cancer
Source: Prostate Cancer Prostatic Dis. 2020 Nov 23;24(2):499–506. doi: 10.1038/s41391-020-00304-1 (PMC8134056; doi:10.1038/s41391-020-00304-1)
Supplement: Supplementary file 1 — Supplementary Material and Methods [file 41391_2020_304_MOESM1_ESM.docx]

## Patient population

Peripheral blood samples from patients with metastatic castration-resistant prostate cancer (mCRPC) were previously collected for circulating tumor cell (CTC) detection and enumeration in the context of a prospective, multicenter, clinical study. The original study design has been described in detail elsewhere (1). In brief, patients were diagnosed antecedently with histologically confirmed prostate adenocarcinoma and suitable for recruitment when starting a new line of endocrine therapy (i.e. abiraterone acetate or enzalutamide) for biochemically defined progressive disease (PD) consistent with Prostate Cancer Clinical Trials Working Group (PCWG3) guidelines (2). The study obtained approval by the ethical committee of the Antwerp University Hospital (registration number: B300201524217) and all patients provided written informed consent. The number of prior chemotherapy or androgen deprivation therapy (ADT) regimens was unrestricted. Samples were collected at treatment initiation (i.e. baseline), in patients who responded for minimally 10-12 weeks, again at 10-12 weeks follow-up, and at disease progression.

## Isolation, enumeration and characterization of EpCAM-positive objects

Enrichment and standard operator-based enumeration of CTCs were performed as previously described (1). All archived immunofluorescence images were retrospectively reanalyzed by means of the open source image analysis tool for Automated CTC Classification, Enumeration and Phenotyping (ACCEPT) (<https://github.com/LeonieZ/ACCEPT>). Upon downloading the images into ACCEPT, the algorithm automatically provides phenotypic measurements for all detected events (larger than four pixels) per fluorescence channel (CK, CD45 and DAPI). These morphological characteristics encompass the eccentricity, perimeter, mean, max and median intensity, size, standard deviation (intensity), mass (sum of all intensities), P2A (perimeter to area) and DNA overlay with the signal in other channels, which were described in detail elsewhere (3-5). Running validated user-defined gating settings (Supplementary Table S1), the software was able to automatically detect and enumerate CTCs and tumor-derived extracellular vesicles (tdEVs), and store the phenotypic characteristics of these EpCAM+ entities in a ready-to-use database (6). Representative thumbnail images of both events can be found in Supplementary Figure S9.

## Statistical analyses

All statistical tests were performed in R (Version 1.1.383), with a two-sided *P*-value <0.05 as being considered as statistically significant. The full data analysis script is made publicly available on GitHub (<https://github.com/StevenVanLaere/ACCEPT>). CTC, tdEV and Shannon Index (SI) levels were compared between defined groups of patients using Mann–Whitney-U or Kruskal-Wallis test, since the variables were not normally distributed according to the ﻿Shapiro-Wilk test. In addition, Levene’s test was used to assess homoscedasticity of the CTC, tdEV and SI distributions between baseline, follow-up and progression subgroups.

Phenotypic features of 19 129 CTCs derived from 331 samples collected from 170 unique mCRPC patients at 3 different time points during treatment were extracted for each immunofluorescence channel. To define phenotypically differing cell types in an unsupervised manner, k-means clustering based on these phenotypic properties acquired through ACCEPT was performed. Different k-means clustering models were established with k-values ranging from 2 to 10. The optimal model was chosen based on a scree-analysis of the total within-cluster sum-of-squares for successive k-values. Building on the grouping of CTCs into a specific cluster (“species”) and the number of such clusters in each sample (“species densities”), the SI was calculated to quantify phenotypic heterogeneity (vegan package) (7). Subsequently, the mean SI (Mean, Range: 0.68, 0-1.59) was used to dichotomize patients with high versus low values.

The overall primary outcome of this study was progression–free survival (PFS), defined as the time between start of therapy and clinical symptomatic progression and/or radiologic progression. The secondary endpoint encompassed overall survival (OS). OS was defined as the time from baseline blood sampling to death from any cause or the last follow-up visit (i.e. censored event). Based on the proportion of events in the entire population (i.e. PFS: 78% and OS: 38%), the total sample size (*n*=143) and the proportion of patients with high numbers of CTCs (36%), tdEVs (26%) or high SI values (50%), our analyses have more then 90% power to detect the reported hazard ratios at a nominal p-value of 5%. The association of CTC and tdEVs counts, phenotype classes, and dynamics during treatment with time-to-event outcome (PFS and OS) in mCRPC patients was evaluated using Kaplan-Meier analysis with log-rank test, Uni- (UV) and multivariable (MV) Cox proportional hazards regression analyses. For the latter, the proportional hazards assumption was checked. Only variables significant for prognostic assessment in UV analysis were included in MV analysis. For cox regression the following covariates were included: age, serum PSA and LDH concentrations, prior treatment with chemotherapy, prior exposure to abiraterone or enzalutamide, CTC count at study entry, and presence of visceral metastases, this combined with a newly defined baseline covariate from this study that was associated with outcome, the SI.

1. De Laere B, Oeyen S, Van Oyen P, Ghysel C, Ampe J, Ost P, et al. Circulating tumor cells and survival in abiraterone- and enzalutamide-treated patients with castration-resistant prostate cancer. Prostate. 2018;78(6):435-45.

2. Scher HI, Morris MJ, Stadler WM, Higano C, Basch E, Fizazi K, et al. Trial Design and Objectives for Castration-Resistant Prostate Cancer: Updated Recommendations From the Prostate Cancer Clinical Trials Working Group 3. J Clin Oncol. 2016;34(12):1402-18.

3. Zeune L, van Dalum G, Decraene C, Proudhon C, Fehm T, Neubauer H, et al. Quantifying HER-2 expression on circulating tumor cells by ACCEPT. PLoS One. 2017;12(10):e0186562.

4. Zeune LL, de Wit S, Berghuis AMS, IJzerman MJ, Terstappen LWMM, Brune C. How to Agree on a CTC: Evaluating the Consensus in Circulating Tumor Cell Scoring. Cytometry A. 2018;93(12):1202-6.

5. de Wit S, Zeune LL, Hiltermann TJN, Groen HJM, Dalum GV, Terstappen L. Classification of Cells in CTC-Enriched Samples by Advanced Image Analysis. Cancers (Basel). 2018;10(10).

6. Nanou A, Coumans FAW, van Dalum G, Zeune LL, Dolling D, Onstenk W, et al. Circulating tumor cells, tumor-derived extracellular vesicles and plasma cytokeratins in castration-resistant prostate cancer patients. Oncotarget. 2018;9(27):19283-93.

7. Oksanen J. Vegan: Community Ecology Package . 2.5-6 ed: R Development Core Team; 2019.
