# Supplementary material for: Automated enumeration and phenotypic characterization of CTCs and tdEVs in patients with metastatic castration resistant prostate cancer
Source: Prostate Cancer Prostatic Dis. 2020 Nov 23;24(2):499–506. doi: 10.1038/s41391-020-00304-1 (PMC8134056; doi:10.1038/s41391-020-00304-1)
Supplement: Supplementary file 2 — Supplementary Figures [file 41391_2020_304_MOESM2_ESM.pdf]

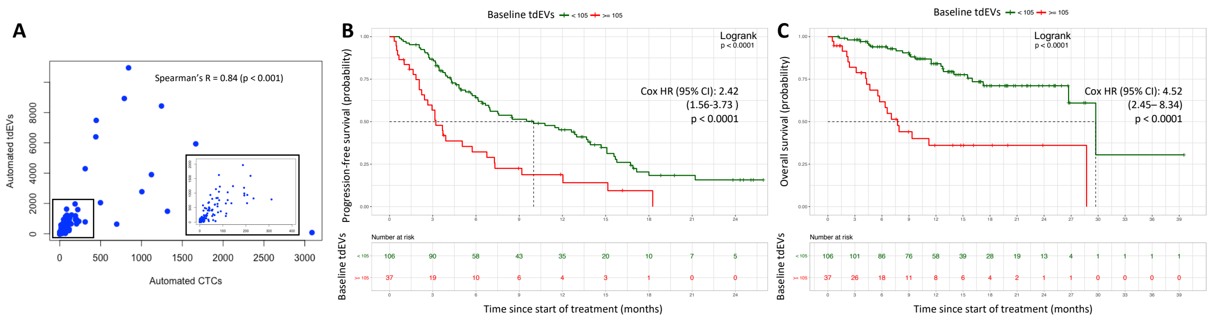

Supplementary Figure S1. Relation between automated CTC and tdEVs counts. *Figure A shows a scatterplot revealing a correlation between CTCs and tdEVs counts, both automatically enumerated. The framed datapoints in panel A are magnified by the inserted figure in that panel. Kaplan-Meier estimates (probabilities) of progression-free (B) and overall survival (C) for baseline tdEVs (C and D) counts of our studied patient cohort. Patients were stratified according to their baseline tdEVs levels < 105 or ≥105 tdEVs/7.5 mL into favorable (green curves) and unfavorable (red curves) groups of patients, respectively. CTCs denotes circulating tumor cells. tdEVs denotes tumor-derived extracellular vesicles. HR denotes hazard ratio. CI denotes confidence interval.*

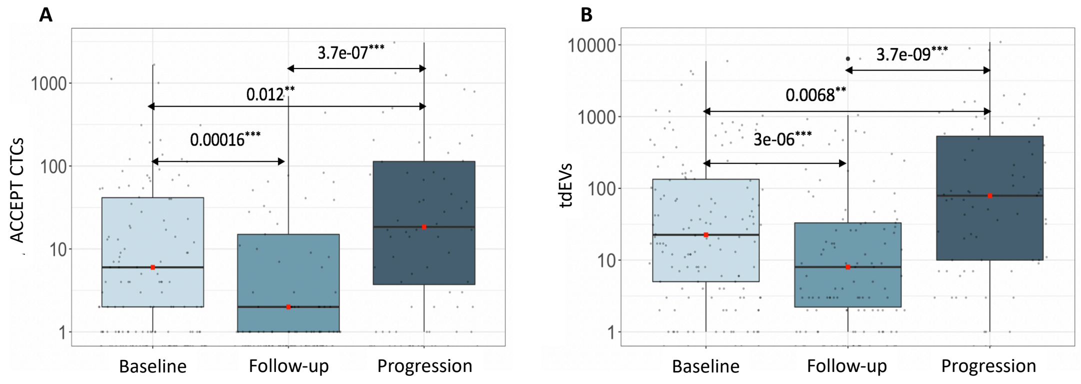

Supplementary Figure S2. Automated CTC (A) and tdEVs (B) counts during treatment with ARSI. *CTCs denotes circulating tumor cells. tdEVs denotes tumor-derived extracellular vesicles. P-values (Kruskal-Wallis) are noted in the plot.*

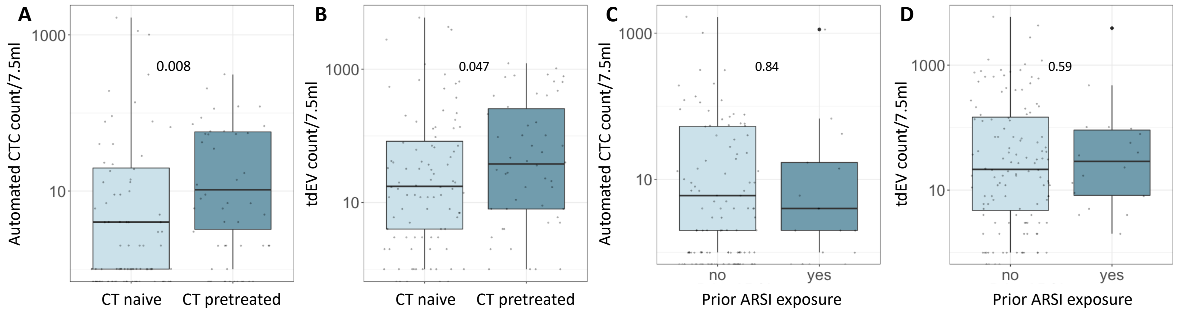

Supplementary Figure S3. The number of automated CTCs (A and C) and tdEVs (B and D) versus prior chemotherapy (A and B) or ARSI (C and D) exposure. *CTCs denotes circulating tumor cells. tdEVs denotes tumor-derived extracellular vesicles. P-values (Mann-Whitney U test) are noted per plot.*

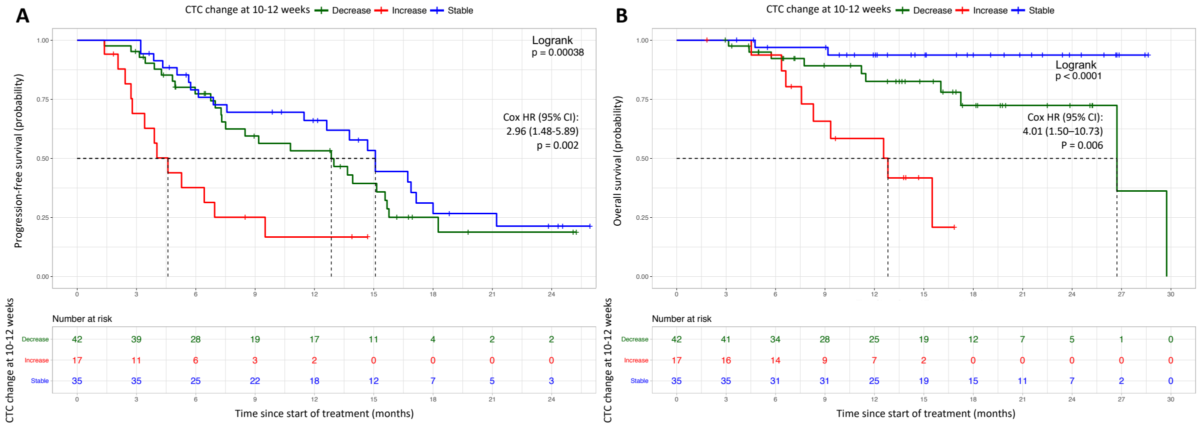

Supplementary Figure S4. Kaplan Meier estimates (probabilities) of progression-free (A) and overall survival (B) for CTC dynamics at 10-12w of follow-up. *Patients were stratified based on their change in CTC levels from baseline to follow-up (green: decrease, red: increase, blue: stable). CTCs denotes circulating tumor cells. HR denotes hazard ratio. CI denotes confidence interval.*

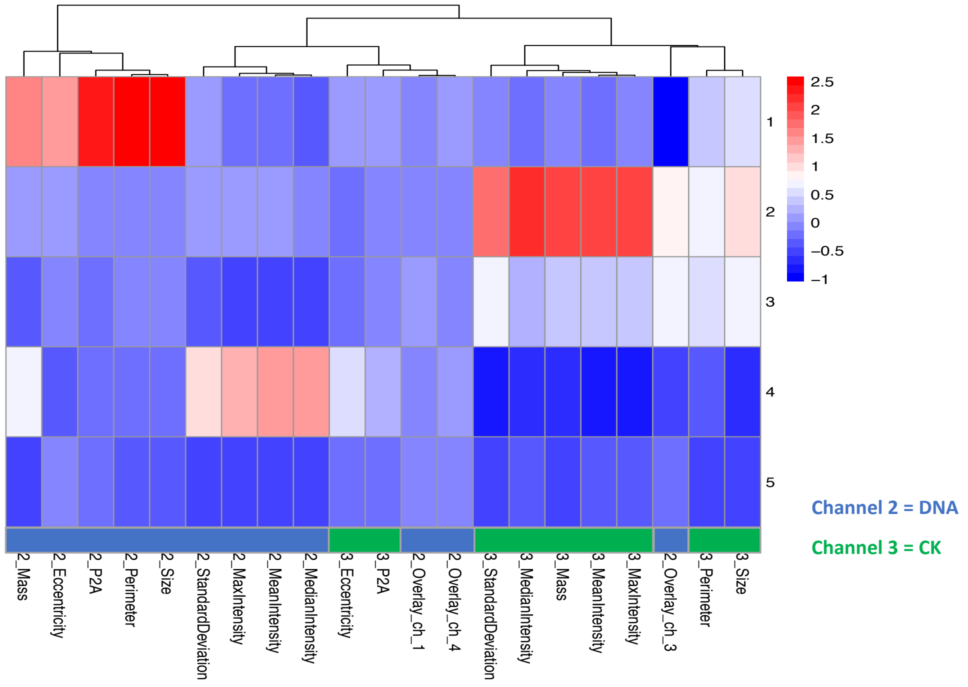

Supplementary Figure S5. Heatmap depicting the phenotypic properties that constitute to the formation of the 5 phenotypic CTC categories. *Each column represents a phenotypic parameter, each row a category made up of these parameters. The colored bar on top of the phenotypic parameters represents the fluorescence channel. The blue color represents the measures acquired for the nuclear channel (DAPI), the green color those for the cytokeratin channel. CTCs denotes circulating tumor cells.*

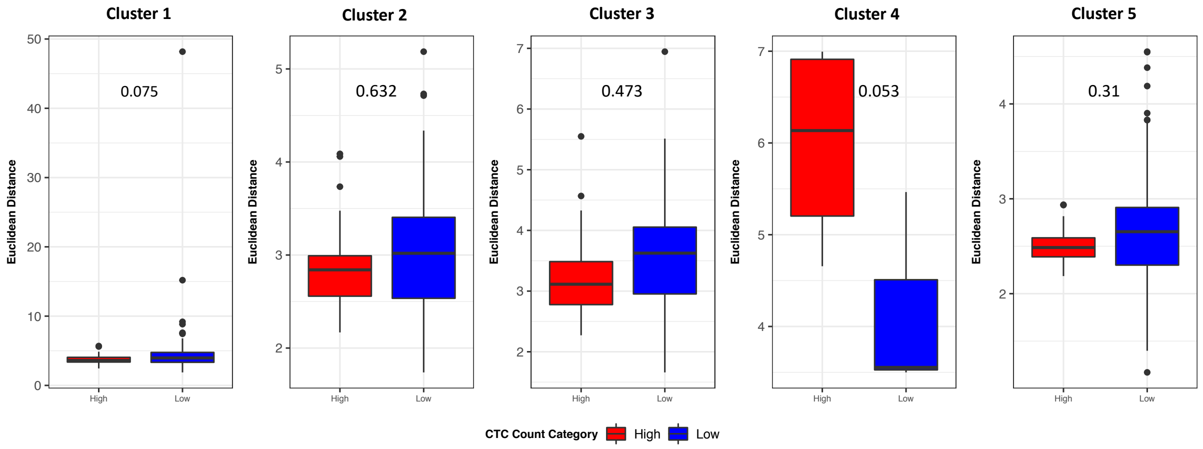

Supplementary Figure S6. The Euclidean distance per cluster for all samples partitioned into a CTC high and low category. *Samples with CTC numbers above the mean number were assigned to the "High" category, samples with numbers below the mean number were assigned to the "Low" category. All P-values are listed in the figure and were corrected for false discovery. CTCs denotes circulating tumor cells.*

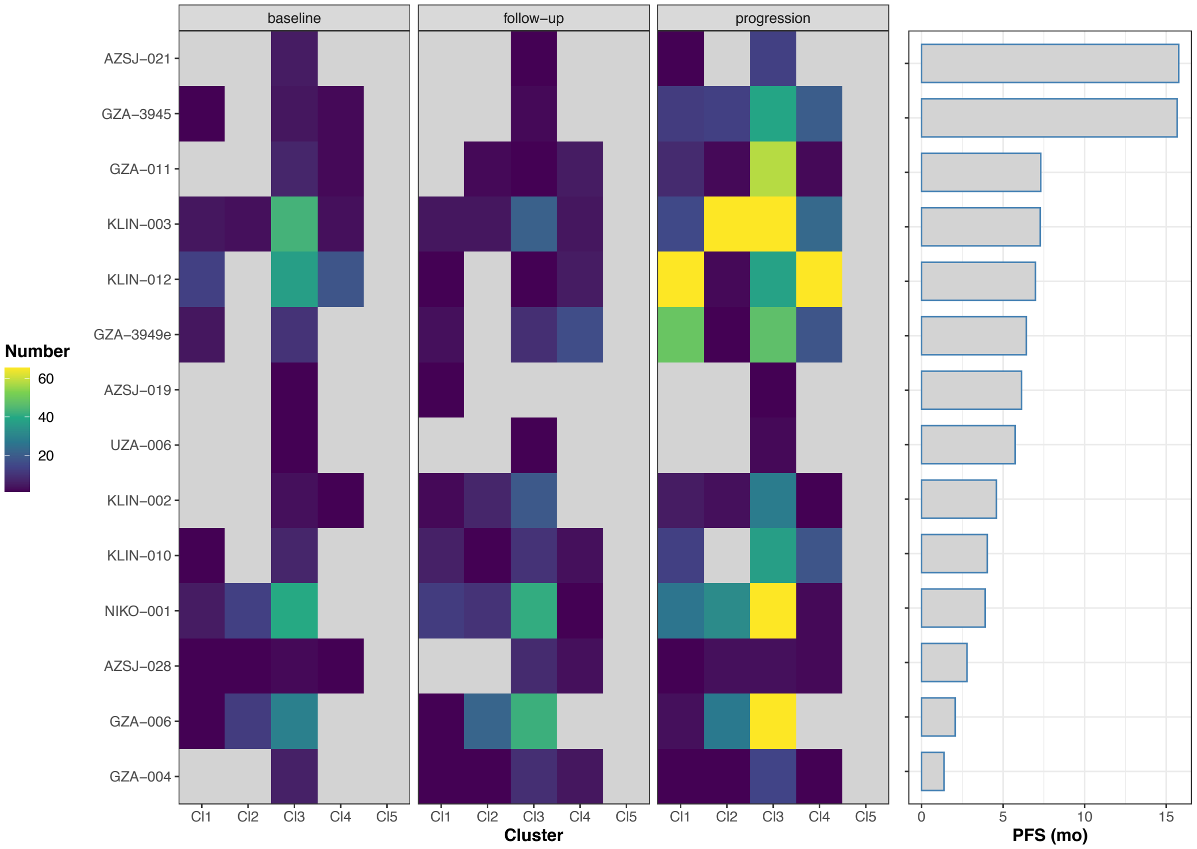

Supplementary Figure S7. Distribution of CTC cluster presence per patient per sample type (baseline, follow-up and progression), represented in heatmap format, ordered by decreasing PFS time, for patients that were CTC positive throughout the entire course of treatment. *The different CTC clusters and patients are shown along the x- and y-axis, respectively. The presence of a CTC cluster in a particular sample is indicated with a color-coded cell and the color illustrates the number of CTC per cluster and sample, with blue to yellow reflecting low to high numbers, respectively. The grey color indicates zero CTC. Patients are ordered according to decreasing PFS intervals (expressed in months), provided in barplot format on the right side of the figure. PFS denotes progression-free survival. CTCs denotes circulating tumor cells.*

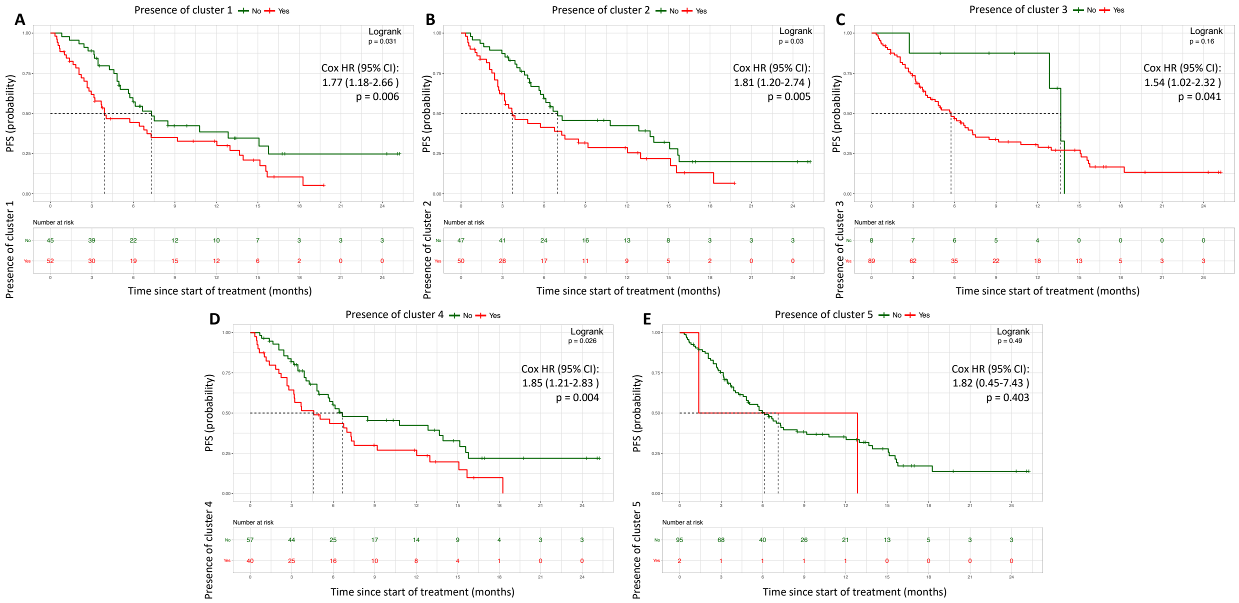

Supplementary Figure S8. Kaplan-Meier estimates (probabilities) of progression-free survival (PFS) for CTC cluster absence or presence. Patients were dichotomized based on the absence or presence of CTC belonging to a specific cluster category (green or red curves, respectively). CTCs denotes circulating tumor cells. HR denotes hazard ratio. CI denotes confidence interval.

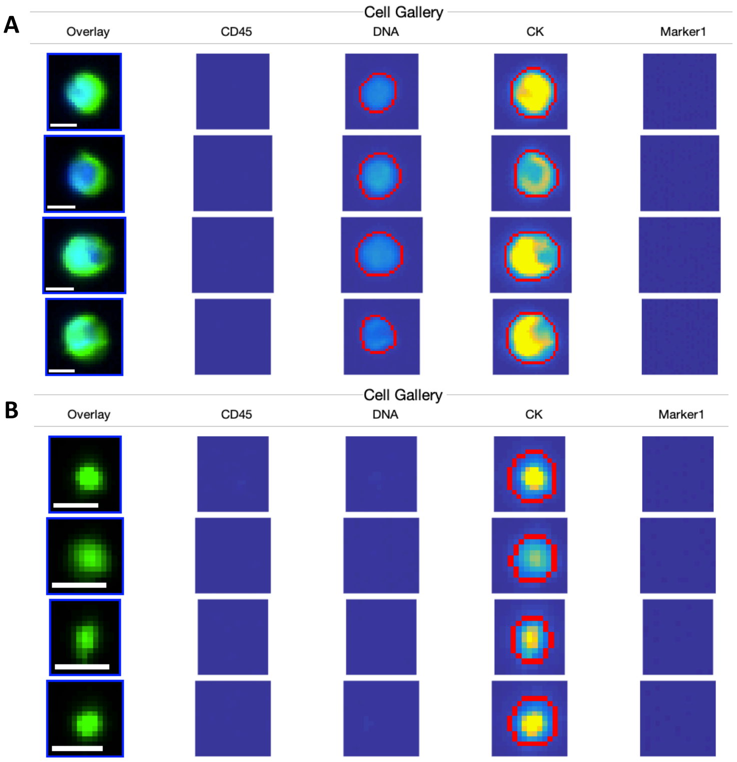

Supplementary Figure S9. Representative thumbnail images of both CTCs (A) and tdEVs (B) by ACCEPT. The red lines define the area where ACCEPT identifies a fluorescence signal for each channel. DNA is represented in blue, cytokeratin (CK) in green and CD45 would be represented by a red signal. The scale bar indicates 6.4 µm.
