# Supplementary material for: Automated enumeration and phenotypic characterization of CTCs and tdEVs in patients with metastatic castration resistant prostate cancer
Source: Prostate Cancer Prostatic Dis. 2020 Nov 23;24(2):499–506. doi: 10.1038/s41391-020-00304-1 (PMC8134056; doi:10.1038/s41391-020-00304-1)
Supplement: Supplementary file 3 — Supplementary Tables [file 41391_2020_304_MOESM3_ESM.docx]

**Supplementary Table S1. Pre-defined ACCEPT gate settings from literature (Nanou, Oncotarget 2018).** CTCs denotes circulating tumor cells. tdEVs denotes tumor-derived extracellular vesicles. Marker 1 and 2 were empty during this study.

|  |  |  |
| --- | --- | --- |
| Event | **Gate setting** |  |
| Single CTC | Mean Intensity CD45 ≤ 5, Mean Intensity DNA > 45, Mean Intensity CK > 60, Mean Intensity Marker 1 ≤ 5, Mean Intensity Marker 2 ≤ 5, 16 ≤ Size CK ≤ 400, DNA Overlay CK > 0.2 |  |
| tdEV | Mean Intensity CD45 ≤ 5, Mean Intensity DNA ≤ 5, Mean Intensity Marker 1 ≤ 5, Mean Intensity Marker 2 ≤ 5, Mean Intensity CK > 60, Max Intensity CK > 90, Size CK ≤ 150, Perimeter CK > 5, Eccentricity CK ≤ 0.8, Perimeter to Area CK ≤ 1 |  |

**Supplementary Table S2. Patient characteristics of studied cohort.** ADT: Androgen deprivation therapy, Radical Px: Radical prostatectomy, RT: Radiation therapy, TURP: Transurethral resection of the prostate, HIFU: high-intensity focused ultrasound, CT: Chemotherapy, LN: Lymph nodes

|  | ***n* (%)** |
| --- | --- |
| **Unique patients** | 170 (100) |
| **Age at registration** (mean in years) | 75 |
| **Tumor stage at diagnosis** |  |
| T1/2 | 44 (26) |
| T3/4 | 43 (25) |
| M1 | 47 (28) |
| Node-positive | 13 (8) |
| Not specified | 23 (14) |
| **Gleason score at diagnosis** |  |
| ≤7 | 72 (42) |
| 8-10 | 75 (44) |
| Not specified | 23 (14) |
| **Primary treatment** |  |
| ADT | 69 (41) |
| Radical Px | 58 (34) |
| RT + ADT | 16 (9) |
| RT | 9 (5) |
| TURP/HIFU | 6 (4) |
| Radical Px + ADT | 6 (4) |
| Radical Px + RT | 4 (2) |
| CT | 1 (0.6) |
| Watchful waiting | 1 (0.6) |
| **Previous chemotherapy** |  |
| Naïve | 106 (62) |
| Pretreated | 64 (38) |
| **Previous ARSI for CRPC** |  |
| No | 147 (86) |
| Yes | 23 (14) |
| **Metastatic burden at start of therapy** |  |
| LN only | 25 (15) |
| Bone only | 65 (38) |
| Bone and LN | 45 (26) |
| Visceral and bone and/or LN | 31 (18) |
| None | 4 (2) |
| **Therapy initiated at baseline** |  |
| Abiraterone Acetate | 120 (71) |
| Enzalutamide | 50 (29) |

**Supplementary Table S3. Laboratory measures at baseline.** This table depicts the prognostic serum marker values and hematology of the studied patient cohort. PSA: prostate-specific antigen, LDH: lactate dehydrogenase, AP: Alkaline phosphatase, Hb: Hemoglobin, RBC: Red blood cells, WBC: White blood cells, NEU: Neutrophils, LYM: Lymphocytes, MON: Monocytes, TROM: Thrombocytes

|  |  |  | |
| --- | --- | --- | --- |
|  | **Median** | **Range** | |
| PSA (*n* = 135; ng/ml) | 22.50 | 0.24-1896 | |
| LDH (*n* = 102; U/L) | 302.5 | 118-2742 | |
| AP (*n* = 106; U) | 97.5 | 34-760 | |
| Hb (*n* = 112; g/dl) | 12.3 | 4.4-15.6 | |
| RBC (*n* = 109; 10e6/mm3) | 4.06 | 1.09-5.43 |  |
| WBC (*n* = 112; 10e3/mm3) | 6.12 | 3-64.82 | |
| NEU (*n* = 101; 10e3/mm3) | 4.51 | 1.7-38.2 | |
| LYM (*n* = 100; 10e3/mm3) | 1.2 | 0.33-14.2 | |
| MON (*n* = 100; 10e3/mm3) | 0.6 | 0.2-11.67 | |
| TROM (*n* = 110; 10e3/mm3) | 219.5 | 68-908 | |
|  |  |  | |

**Supplementary Table S4. Uni- and multivariable Cox proportional hazards regression analysis of progression-free (A) and overall (B) survival based on tdEV counts, previous and newly defined baseline characteristics.** PFS and OS denote progression-free and overall survival, respectively. HR denotes hazard ratio. CI denotes confidence interval. tdEV denotes tumor-derived extracellular vesicle. PSA denotes prostate-specific antigen. LDH denotes lactate dehydrogenase. SI denotes Shannon index *P values from Wald test of Z statistic.

|  |  | **Univariable** | | **Multivariable** | |
| --- | --- | --- | --- | --- | --- |
|  |  | **Cox proportional hazard** | | **Cox proportional hazard** | |
| **Variable** | **Categories** | **HR (95% CI)** | ***P**** | **HR (95% CI)** | ***P**** |
| **(A) PFS** |  |  |  |  |  |
| Age | Continuous | 1.018 (0.993-1.043) | 0.159 |  |  |
| Chemotherapy status | Naïve vs pretreated | 2.601 (1.729-3.912) | **<0.0001** | 2.763 (1.422-5.370) | **0.003** |
| Prior ARSi exposure | No vs yes | 1.715 (1.011-2.909) | **0.045** | 1.041 (0.447-2.427) | 0.926 |
| Metastases at start of study | Non-visceral/nodal vs visceral | 2.402 (1.459-3.953) | **0.001** | 1.000 (0.512-1.953) | 0.999 |
| Baseline PSA | Continuous | 1.002 (1.001-1.002) | **<0.0001** | 1.001 (1.000-1.002) | 0.094 |
| Baseline LDH | Continuous | 1.001 (1.001-1.002) | **<0.0001** | 1.000 (1.000-1.001) | 0.977 |
| Baseline tdEVs (/7.5mL) | Continuous | 1.001 (1.001-1.001) | **<0.0001** | 1.001 (1.001-1.001) | **<0.0001** |
| Baseline SI | Continuous | 1.918 (1.196-3.075) | **0.007** | 1.500 (0.716-3.146) | 0.283 |
| **(B) OS** |  |  |  |  |  |
| Age | Continuous | 1.008 (0.971-1.048) | 0.671 |  |  |
| Chemotherapy status | Naïve vs pretreated | 3.567 (1.875-6.786) | **0.0001** | 2.762 (1.280-5.960) | **0.010** |
| Prior ARSi exposure | No vs yes | 1.853 (0.884-3.884) | 0.102 |  |  |
| Metastases at start of study | Non-visceral/nodal vs visceral | 2.879 (1.509-5.493) | **0.001** | 1.610 (0.752-3.447) | 0.220 |
| Baseline PSA | Continuous | 1.002 (1.001-1.003) | **<0.0001** | 1.001 (1.000-1.003) | 0.077 |
| Baseline LDH | Continuous | 1.001 (1.0004-1.002) | **0.001** | 1.000 (0.999-1.001) | 0.426 |
| Baseline tdEVs (/7.5mL) | Continuous | 1.001 (1.001-1.002) | **<0.0001** | 1.001 (1.000-1.002) | **0.0003** |
| Baseline SI | Continuous | 3.034 (1.479-6.228) | **0.002** | 1.472 (0.580-3.738) | 0.416 |

**Supplementary Table S5. Uni- and multivariable Cox proportional hazards regression analysis of progression-free (A) and overall (B) survival based on CTC counts, previous and newly defined baseline characteristics in a subset of patients that were both ARSi- and chemotherapy-naïve at study entry (n = 87).** PFS and OS denote progression-free and overall survival, respectively. HR denotes hazard ratio. CI denotes confidence interval. CTC denotes circulating tumor cells. PSA denotes prostate-specific antigen. LDH denotes lactate dehydrogenase. SI denotes Shannon index *P values from Wald test of Z statistic.

|  |  | **Univariable** | | **Multivariable** | |
| --- | --- | --- | --- | --- | --- |
|  |  | **Cox proportional hazard** | | **Cox proportional hazard** | |
| **Variable** | **Categories** | **HR (95% CI)** | ***P**** | **HR (95% CI)** | ***P**** |
| **(A) PFS** |  |  |  |  |  |
| Age | Continuous | 1.034 (0.999-1.071) | 0.060 |  |  |
| Metastases at start of study | Non-visceral/nodal vs visceral | 1.926 (0.812-4.572) | 0.137 |  |  |
| Baseline PSA | Continuous | 1.001 (1.001-1.002) | **0.0003** | 1.001 (1.000-1.002) | **0.003** |
| Baseline LDH | Continuous | 1.001 (1.001-1.002) | **0.0001** | 1.000 (1.000-1.001) | 0.342 |
| Baseline CTC (/7.5mL) | Continuous | 1.003 (1.002-1.004) | **<0.0001** | 1.003 (1.001-1.004) | **0.006** |
| Baseline SI | Continuous | 1.745 (0.919-3.313) | 0.089 |  |  |
| **(B) OS** |  |  |  |  |  |
| Age | Continuous | 1.073 (0.988-1.166) | 0.095 |  |  |
| Metastases at start of study | Non-visceral/nodal vs visceral | 5.389 (1.574-18.455) | **0.007** | 3.391 (0.664-17.321) | 0.142 |
| Baseline PSA | Continuous | 1.002 (1.001-1.003) | **0.0009** | 1.002 (1.001-1.004) | **0.003** |
| Baseline LDH | Continuous | 1.001 (1.0003-1.002) | **0.006** | 1.000 (0.998-1.001) | 0.642 |
| Baseline CTC (/7.5mL) | Continuous | 1.003 (1.002-1.005) | **<0.0001** | 1.004 (1.000-1.008) | 0.069 |
| Baseline SI | Continuous | 3.368 (1.042-10.885) | **0.043** | 1.436 (0.320-6.432) | 0.637 |

**Supplementary Table S6. Uni- and multivariable Cox proportional hazards regression analysis of progression-free (A) and overall (B) survival based on tdEV counts, previous and newly defined baseline characteristics in a subset of patients that were both ARSi- and chemotherapy-naïve at study entry (n = 87).** PFS and OS denote progression-free and overall survival, respectively. HR denotes hazard ratio. CI denotes confidence interval. tdEV denotes tumor-derived extracellular vesicle. PSA denotes prostate-specific antigen. LDH denotes lactate dehydrogenase. SI denotes Shannon index *P values from Wald test of Z statistic.

|  |  | **Univariable** | | **Multivariable** | |
| --- | --- | --- | --- | --- | --- |
|  |  | **Cox proportional hazard** | | **Cox proportional hazard** | |
| **Variable** | **Categories** | **HR (95% CI)** | ***P**** | **HR (95% CI)** | ***P**** |
| **(A) PFS** |  |  |  |  |  |
| Age | Continuous | 1.034 (0.999-1.071) | 0.060 |  |  |
| Metastases at start of study | Non-visceral/nodal vs visceral | 1.926 (0.812-4.572) | 0.137 |  |  |
| Baseline PSA | Continuous | 1.001 (1.001-1.002) | **0.0003** | 1.001 (1.000-1.002) | **0.010** |
| Baseline LDH | Continuous | 1.001 (1.001-1.002) | **0.0001** | 1.001 (1.000-1.001) | 0.060 |
| Baseline tdEVs (/7.5mL) | Continuous | 1.001 (1.001-1.001) | **<0.0001** | 1.001 (1.000-1.001) | **0.004** |
| Baseline SI | Continuous | 1.745 (0.919-3.313) | 0.089 |  |  |
| **(B) OS** |  |  |  |  |  |
| Age | Continuous | 1.073 (0.988-1.166) | 0.095 |  |  |
| Metastases at start of study | Non-visceral/nodal vs visceral | 5.389 (1.574-18.455) | **0.007** | 3.859 (0.781-19.063) | 0.098 |
| Baseline PSA | Continuous | 1.002 (1.001-1.003) | **0.0009** | 1.002 (1.001-1.004) | **0.003** |
| Baseline LDH | Continuous | 1.001 (1.0003-1.002) | **0.006** | 0.999 (0.997-1.002) | 0.637 |
| Baseline tdEVs (/7.5mL) | Continuous | 1.001 (1.001-1.002) | **<0.0001** | 1.002 (0.999-1.004) | 0.163 |
| Baseline SI | Continuous | 3.368 (1.042-10.885) | **0.043** | 1.234 (0.279-5.461) | 0.782 |
